# Design and Reliability of a Novel Heel Rise Test Measuring Device for Plantarflexion Endurance

**DOI:** 10.1155/2014/391646

**Published:** 2014-04-30

**Authors:** Amy D. Sman, Claire E. Hiller, Adam Imer, Aldrin Ocsing, Joshua Burns, Kathryn M. Refshauge

**Affiliations:** ^1^Discipline of Physiotherapy, Faculty of Health Sciences, The University of Sydney, Cumberland Campus C42, P.O. Box 170, Lidcombe, NSW 1825, Australia; ^2^Institute for Neuroscience and Muscle Research, The Children's Hospital at Westmead, Locked Bag 4001, Westmead, NSW 2145, Australia

## Abstract

*Background*. Plantarflexion results from the combined action of the soleus and gastrocnemius muscles in the calf. The heel rise test is commonly used to test calf muscle endurance, function, and performance by a wide variety of professionals; however, no uniform description of the test is available. This paper aims to document the construction and reliability of a novel heel rise test device and measurement protocol that is suitable for the needs of most individuals. *Methods*. This device was constructed from compact and lightweight materials and is fully adjustable, enabling the testing of a wide variety of individuals. It is easy to assemble and disassemble, ensuring that it is portable for use in different settings. *Findings*. We tested reliability on 40 participants, finding excellent interrater reliability (ICC_2,1_ 0.97, 95% CI: 0.94 to 0.98). Limits of agreement were less than two repetitions in 90% of cases and the Bland-Altman plot showed no bias. *Interpretation*. We have designed a novel, standardized, simple, and reliable device and measurement protocol for the heel rise test which can be used by researchers and clinicians in a variety of settings.

## 1. Introduction

Plantarflexion is achieved through the combined action of the soleus and gastrocnemius muscles, located in the calf [1, 2]. The gastrocnemius and soleus muscles share the Achilles tendon, the thickest and strongest human tendon, and are also known as the triceps surae [2]. The triceps surae is responsible for 80% of plantarflexion force [1–3]. Combined, the triceps surae stabilises the foot in weight bearing and produces a plantarflexion moment at toe-off, essential for forward momentum during gait [1, 2]. Therefore, sufficient plantarflexion strength and endurance are essential for basic mobility, such as walking and running [2, 4].

The heel rise test, also commonly described as heel raise test or calf rise test, was first developed in the 1940s [4, 5] to assess the function of the calf muscle-tendon unit and is now widely used by clinicians and researchers across disciplines. The test has commonly been used in neurology, cardiology, gerontology, orthopaedics, and sports medicine [5]. Plantarflexion strength and endurance are often impaired after lower limb injury, especially after injury to the Achilles tendon [1, 6]. The heel rise test is commonly used not only during the initial assessment of these injuries, but also during rehabilitation to quantify treatment outcomes [5]. The test is often described as a test of calf muscle endurance, strength, fatigue, function, and performance [5, 7, 8]. It involves standing on one leg and rising and lowering the body by lifting the heel off the ground and then lowering it while maintaining a straight knee. The task is repeated without a break until the participant cannot complete it correctly or complains of pain or fatigue in the calf muscles [1, 4, 9]. The number of heel rises a participant can achieve is then recorded. The research literature commonly recommends 25 heel rises as the target norm of clinical performance for healthy subjects; however, much higher and lower values ranging from 7 to 48 have also been suggested in both research literature and musculoskeletal textbooks [5].

A systematic review by Hébert-Losier et al. [5] investigated the test variables and concluded that although this test is widely used in various health professions as a recommended assessment and rehabilitation tool, there is no uniform description of the test. The wide range of reported normative values in the literature may reflect the lack of standardization of the test. This further emphasises the need that the development of a standardized, reliable heel rise test is important for researchers and clinicians alike [1].

Research studies using the heel rise test commonly use customised devices developed to standardize the test and monitor test variables; however, these devices were often extremely complex, prohibiting use in everyday clinical practice or inadequately controlled confounding variables. In one study [10], a device was constructed that consisted of a box with an incline, a weight belt attached to the waist of the participant, and a linear encoder attached to the heel of the shoe to measure plantarflexion endurance. The linear encoder, connected to a computer measuring system, measured the time and length of the heel displacement of the heel rise. A second study [11] used a device with a light beam attached to vertical rods at a fixed height of 5 cm above the heel. The device emitted an audible click when the participant's heel passed the 5 cm height when rising onto the toes to raise the body. However, no feedback was provided to participants about the actual height of each heel rise and therefore a maximum or standardized heel rise was not necessarily achieved. A further disadvantage of this device was that the height was not adjustable and therefore could not allow individualized testing. Additionally, the device required an electrical current to function. A third study [12] used a simple, portable device that positioned a rod horizontally across the foot for participants to touch with the anterior aspect of the arch of the foot when the heel was raised. Some adjustability was allowed by four preset holes in the device in which the rod was placed to enable selection of four different heights. Although the device allowed for some ability to individualize the test, adjustability was incomplete. Furthermore, the safety of placing the rod at the front of the foot is questionable: in the event that an individual lost balance during the test, the rod could prevent stepping off the device with ease.

Clinically, the heel rise test is often employed for assessment and rehabilitation purposes without using a device at all. This may possibly be due to the complexity of devices currently available. However, a standardized device and protocol that is suitable for all individuals is essential to monitor and replicate the heel rise test with consistent outcomes [1, 5]. When the aim is to use the device in clinical settings, it should be simple, cheap, reliable, and clinically accessible [5]. A universally accepted standardization of the heel rise test and consequently a device that allows for standardized, reliable, and individualized evaluation protocols is not currently available. The Ankle Measure for Endurance and Strength (AMES) device (IP Australia; innovation patent application number AU2012101251) and measurement protocol was created to provide the platform for such results. This paper aims to document the construction and reliability of the device.

## 2. Methods

### 2.1. Construction of the Device

**Figure 1:**
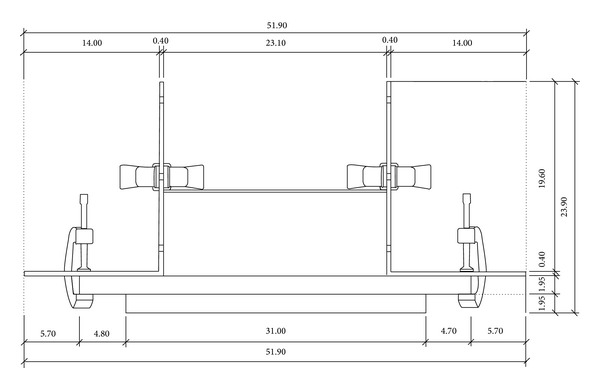
Front view of the Ankle Measure for Endurance and Strength (AMES) device.

**Figure 2:**
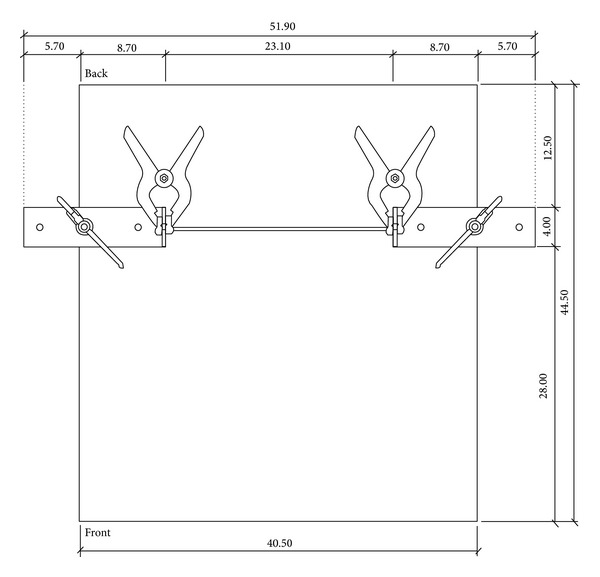
Top view of the AMES device.

The construction of the AMES device is shown in Figures 1 and 2. We used a 44.5 cm × 40.5 cm (*L* × *W*) wooden platform as the base. To the bottom we glued two small wooden blocks each of 31 cm × 4.2 cm (*L* × *W*) to lift the platform slightly off the ground and to allow fixation of the other parts to the platform.

On top of the platform, two medium sized “L”-brackets were placed parallel to each other on both sides, spaced 23.1 cm apart and 12.5 cm from the back of the platform. The setting of the brackets as illustrated in Figures 1 and 2 has proven to fit the foot length and width of all tested individuals. However, the brackets can be moved forwards, backwards, or wider apart to accommodate individuals' foot length and width. The L-brackets were secured onto the platform with two small “G”-clamps of 12 × 8 cm (*L* × *W*).

A 12 mm thick elastic band, on which the participant stands, was placed horizontally between the L-shape brackets and was held in place by two small spring clamps. The elastic band was 30 cm long and cut from a 4-meter strip. The L-shape brackets, the elastic band, the G-clamps, and the spring clamps are adjustable so that the device is able to fit the needs of a wide variety of individuals. The spring clamp facilitates the sliding of the elastic band up and down the L-brackets to cater for each individual's maximum heel rise height. Adjustment of the spring clamps and the elastic band was performed while the individual was standing on the platform and performing a heel rise. The platform, the small wooden blocks, the L-shape brackets, the G-clamps, the spring clamps, and the elastic band were all constructed from compact and lightweight materials and attached to each other but all were removable to facilitate easy repeated assembly and disassembly of the device. The total price of the current device and all its components was approximately $25.00 USD.

### 2.2. Testing Set-Up Protocol

Before performing the test, the device was adjusted to the individual's maximum heel rise as follows.

**Figure 3:**
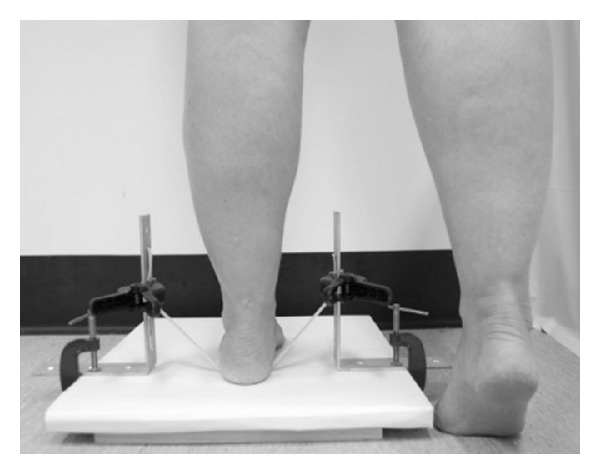
Testing set-up (step 1).

**Figure 4:**
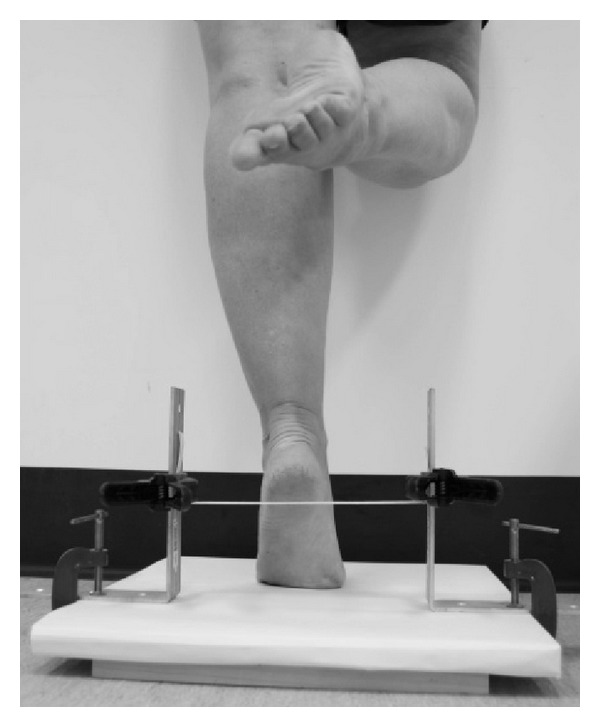
Testing set-up (steps 2–5).

1. The participant placed the heel, barefoot, on the elastic band between the L-brackets with the individual's foot pointing to the front of the platform (Figure 3).
2. The participant performed a maximum heel rise, with extended knee, with the nontesting leg flexed and suspended in the air and the fingertips of one hand on the wall for balance (Figure 4).
3. The examiner adjusted the elastic band by sliding the spring clamps up or down until the elastic band was just clear of the heel and in a horizontal position. The participant lowered their heel back onto the platform.
4. The participant performed another heel rise to confirm their maximum heel rise; for example, the participant cleared the elastic band on each heel rise during the test.
5. Lastly, the test was conducted with the participant rising and lowering their heel to the beat of a metronome until the participant can no longer perform a heel rise or fails to perform a technically correct heel rise (maximum heel raise with extended knee, clearing the elastic band, and only fingertips of one hand on the wall for balance) on two consecutive occasions.

### 2.3. Reliability of the Device

Following the design of the device, we tested it for reliability.

#### 2.3.1. Participants

The study was performed at the University of Sydney in the Arthritis and Musculoskeletal Research Lab. We recruited participants through advertisements on university noticeboards. A total of 40 participants who met the inclusion criteria for the study were enrolled. Inclusion criteria were as follows: (1) healthy adults over 18 years and (2) no current ankle injury or chronic ankle pain. The only exclusion criterion was any present condition, such as vestibular problems or current lower limb injury that would affect balance ability. Participants were asked if they had been involved in excessive exercise during the 72 hours prior to the test occasion, other than their regular exercise. Ethics approval was obtained from the Human Research Ethics Committee at the University of Sydney (Protocol number 07-2011/13973) and all participants gave their written informed consent prior to commencement of the study.

#### 2.3.2. Randomization

Participants performed one trial and the order of test leg was randomly assigned using a web-based randomization program; http://www.randomizer.org/.

#### 2.3.3. Blinding

The test was performed once and observed by two blinded raters (A.I/A.O). A screen separated the raters from each other to minimise bias. For reliability testing purposes, an independent examiner (A.S), shielded from the raters by a second screen, was present to provide the participants with feedback and correct any errors of technique that occurred during the trial. This feedback was given without verbal cues so that the raters were not alerted to potential errors in performance and thus minimise confounding. The independent examiner would provide a tap on the independent examiner's own ankle indicating participants should rise higher on the next heel rise, a tap on the knee indicated participants should keep their knee straight during the next heel rise, and a tap on the hand indicated they were leaning too much into the wall. Participants were instructed to keep eye contact with the independent examiner throughout the test.

#### 2.3.4. Methods

All participants completed a questionnaire including demographic information and medical history related to their ankle and knee to ensure they had no underlying ankle or knee problems.

Participants performed the single leg heel rises barefoot while maintaining an extended knee of the test leg throughout the trial with the nontest leg flexed and suspended in the air. Participants performed a maximum heel rise [1, 5], clearing the elastic band, and then lowered their heel to the platform at the beat of the metronome. As used in previous research by Haber et al. [12], we chose a rate of 46 beats/min (23 heel rises/min) as set by a metronome. On the sound of the first beat of the metronome, the participant lifted the heel and on the following beat lowered the heel. Participants continued the heel rises and were encouraged to do so by the independent examiner until they could no longer perform a technically correct heel rise. For the duration of the trial, participants were allowed to place the fingertips of one hand on the wall for balance.

The raters would stop counting when the participant on 2 consecutive occasions

- could no longer achieve the maximal rise (clear the elastic band) and/or
- placed too much weight on the wall (i.e., hip flexion) and/or
- flexed their knee during the movement and/or
- missed a beat of the metronome and/or
- wished to stop.

One rater (A.O) adjusted the device for each participant to ensure that the same method was used for all participants and to eliminate variability in the setting of the device that could affect the results. The elastic band, 30 cm long, was replaced after every 5 participants to ensure similar elasticity for each participant.

#### 2.3.5. Data Analysis

Data analysis was performed using IBM SPSS statistics 19.0. Data were tested for normality using the Kolmogorov-Smirnov with Lilliefors significance correction and data are reported accordingly. Interrater reliability was calculated by intraclass correlation coefficient (ICC) and limits of agreement. ICCs were calculated using the two-way random effects model (ICC_2,1_) with 95% confidence intervals. Standard error of measurement (SEM) was calculated using the method described by Portney and Watkins [13]; for example, $\text{SEM}=\text{SD}\sqrt{1}-\text{ICC}$, where SD is the standard deviation of the set of observed test scores. A Bland-Altman plot was constructed to determine if there was bias in the measures.

#### 2.3.6. Results

We enrolled 40 participants in the study and all participants completed testing. Their demographic variables are presented in Table 1. The mean age was 24 years and 32 participants were aged between 21 and 23 years. Twenty-one participants reported a history of foot and ankle injury, one reported a history of calf injury, and ten reported a history of knee injury. Of the foot and ankle injuries, 17 were due to ankle sprain. The mean number of heel rises in this healthy population was 23 (SD 13.3) repetitions. Within our cohort, four individuals completed more than 45 repetitions and most individuals completed fewer than 25 repetitions (63%). There was no noticeable difference between males and females. Most trials required approximately 2-3 minutes to complete and no adverse events were reported as a result of the heel rise test or the device.

**Figure 5:**
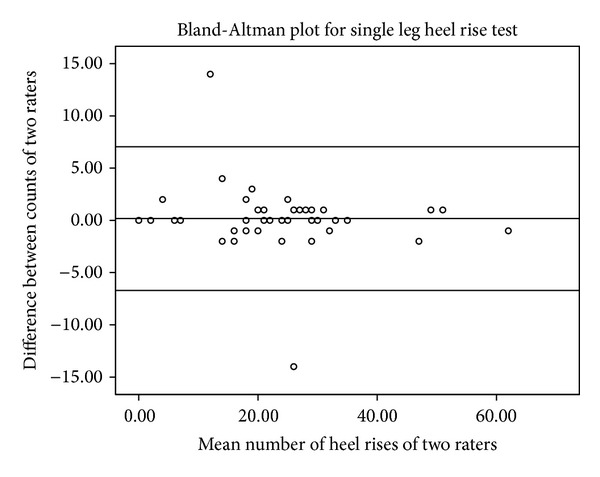
Bland-Altman plot.

Excellent interrater reliability was found between the two trials (ICC_2,1_ 0.97, 95% CI: 0.94 to 0.98). The mean difference between the trials was 0.15 repetitions, the standard error of measurement was 2.4 repetitions (SEM% = 10.4%), and the limits of agreement were less than two repetitions in 90% of the cases (Table 2). The Bland-Altman plot (Figure 5) showed no bias.

## 3. Discussion

The heel rise test is a common test of function of the calf muscle-tendon unit and is used as a test to assess calf muscle endurance and strength [5, 7, 8]. However, until now, there was no standardized device or method to measure calf muscle endurance. The device described in this paper aimed to address the limitations of previous devices, such as the use of computers and linear encoders [10], nonadjustable heights [11, 12], light beams [11], electrical currents [11], and safety of the device [12].

The current study found that, compared to other devices, the current device is safe, cheap, easy to use, and portable. The height of the device is fully adjustable, the elastic band is placed underneath the heel, and the device does not require additional computers or an electrical current to function. The device has excellent interrater reliability and is cheap, portable, and easy and quick to use. The heel rise test can be used in a broad range of settings and has previously been employed during the initial assessment following injury to evaluate treatment progress [5] and to determine return to sport readiness following ankle syndesmosis injury [14, 15] and in research studies investigating risk factors for ankle injury [16]. The device described in this paper may be used as a standardized tool for these purposes in a broad range of clinical populations.

Previous literature reported a wide range of norms using the heel rise test in healthy populations [5] possibly due to the lack of standardization of the test. In a young and healthy population, using the current standardized device and protocol, the mean number of maximum heel rises performed was 23 (SD 13.3). This value may be utilised in the clinic and future research studies as a clinical reference. Although research suggested that the number of heel rises performed varies with age and sex [17], we did not find any notable differences between genders in our study. We were unable to investigate the influence of age on the number of heel rises performed with the current device as the majority of our participants were aged between 21 and 23. Future, larger studies may use this standardized device and protocol to establish norms across the life span.

The current device may be further developed to improve the applicability of the device. Firstly, the current device has no fixation at the front of the foot, which may cause the foot to slide forward during the testing and possibly influence the maximum heel rise height a participant can achieve. Although there were no adverse events due to the device or the tests, the L-shaped brackets may be replaced with curved brackets to minimize risk of injury due to the sharp edges. A limitation to the current reliability testing was the use of an independent examiner to provide nonverbal cues. In daily practice, verbal cues are given by the clinician. It is unknown if this method has influenced the results in any way; however, due to the research design we were unable to change this. Finally, the intrarater reliability has not yet been established, but may be conducted to investigate reproducibility of the test.

## 4. Conclusion

The heel rise test is widely used for clinical assessment and subsequent decision-making regarding rehabilitation progression by a variety of health professionals; however, to date there has been no standardized heel rise test device that is reliable, portable, and easy to use. We have constructed and tested a novel, standardized, and simple device with a standardized measurement protocol for the heel rise test and demonstrated its excellent reliability.

## Figures and Tables

**Table 1:** Participant characteristics (*N* = 40).

Gender	
Female : male	19 : 21
Age (yrs)	
Mean (SD)	24 (6.2)
Height (cm)	
Mean (SD)	174 (12.3)
Weight (kg)	
Mean (SD)	68 (9.3)
Involved in regular exercise^‡^	
Yes : no	5 : 34
History of foot/ankle injury	
Yes : no	23 : 17
History of calf injury	
Yes : no	1 : 39
History of knee injury	
Yes : no	10 : 30

^‡^Missing data for one participant.

**Table 2:** Limits of agreement (*N* = 40).

Difference between raters	Frequency	Valid percent	Cumulative percent
0	14	35.0	35.0
1	14	35.0	70.0
2	8	20.0	90.0
3	1	2.5	92.5
4	1	2.5	95.0
14	2	5.0	100

Total	40	100	
